# Robotic Technology in the Care of Older Persons: A Cross-Sectional National Survey Among Adults in Switzerland

**DOI:** 10.1093/geroni/igaf051

**Published:** 2025-06-17

**Authors:** Tenzin Wangmo, Yi Jiao (Angelina) Tian, Delphine Roulet Schwab, Andrea H Meyer

**Affiliations:** Institute for Biomedical Ethics, University of Basel, Basel, Switzerland; Institute for Biomedical Ethics, University of Basel, Basel, Switzerland; La Source, School of Nursing Sciences, HES-SO University of Applied Sciences and Arts of Western Switzerland, Lausanne, Switzerland; Department of Psychology, Division of Clinical Psychology and Epidemiology, University of Basel, Basel, Switzerland

**Keywords:** Caregiving, Companionship, Ethics, Human care, Robots

## Abstract

**Background and Objectives:**

Robotic technologies will likely be part of the caregiving needs for older adults in the future. In this study, we assessed the acceptance of several robotic functions among a representative sample of adults in Switzerland and tested (a) the acceptance of different robotic functions, and (b) explored how different sets of predictors explained variance in the acceptance of 2 robotic functions: (a) “robots for assistive support” and (b) “robots for companionship.”

**Research Design and Methods:**

A survey was administered to a randomly selected group of adults from the 3 official linguistic regions of Switzerland using computer-aided-telephone-interviews. Data obtained were weighted for the Swiss adult population and analyzed using descriptive statistics, multilevel modeling, and sequential regression analysis.

**Results:**

A total of 1,211 adults responded to the survey. Acceptance was higher for using “robots for assistive support” than “robots for companionship,” with no significant statistical difference between linguistic regions. Usefulness of robotic functions in reducing caregiving stress explained the most variance in our model for both outcome variables. External predictors such as the fear of robots and the fear that robots will replace human care explained the least amount of variance.

**Discussion and Implications:**

When robots are used in the care of older adults, user adoption is likely to be positive when the end-users (older persons and their caregivers) perceive that their use meaningfully reduces caregiving stress. More research is needed to further test the role of external factors for technology adoption, especially those that touch the notion of human contact.

**Translational Significance:** The study found that there was greater acceptance when using robots for assistive purposes such as helping with household chores than having robots as listening and talking companions. Interestingly, fear of robotic technology did not have as much impact on acceptance as predicted. Further research is needed to tease out which type of care can be outsourced to robots and those that should be performed by caregivers only. We also need to study those who refuse the use of potentially beneficial technology despite existing care needs.

## Robots in Caregiving

Many types of robots are available or are under development to support the provision of care to older adults (Cavallo et al., 2018; Portugal et al., 2019; Robinson et al., 2014). These robots may allow aging-in-place by assisting older persons in their personal tasks, helping them maintain their health, and provide social interactions. The fulfillment of these tasks differentiates robots into assistive robots and socially assistive robots (Bemelmans et al., 2012). The hope is that robots will address concerns of fewer professional and family caregivers available to support older persons. In light of the demographic development and healthcare situation in many Western countries, adequately meeting the care needs of older adults is a widespread challenge. Thus, robotic “care” is sought to ameliorate the shortcomings in the family and healthcare spheres, as underscored by a study from Japan (Elsy, 2020). With the pace of current technological development, it is likely that the deployment of robots to help older persons in different spheres of their daily lives might become a reality in the future.

### Understanding Technology Acceptance

Decades ago, acceptance models for information technology systems were developed (Davis, 1987; Venkatesh et al., 2003). Concerning the acceptance of robotic technology, the Almere model (Heerink et al., 2010) concluded that the intention to use technology among older adults was predicted by attitudes such as perceived usefulness, ease of use, enjoyment, and trust. Another scholarship alluding to technology acceptance is the Elderadopt model (Golant, 2017), which proposed that in addition to the variables (e.g., usefulness), technology acceptance depends on (a) alternatives available, (b) appraisal of all technological options, (c) the severity of user’s need, and (d) internal as well as external information that affect user’s understanding of the technology. Finally, Thielke (2012) stated that technology responding to a set of basic needs will find greater acceptance.

At the individual level, for instance, the models of acceptance (Heerink et al., 2010; Venkatesh et al., 2003) report gender and age as moderating variables, with men and younger persons more likely to use a technology if it produces any benefits, whereas women were more influenced by the ease of using a technology (Venkatesh et al., 2003). Several studies included in Huang and Oteng’s (2023) systematic review concluded that having health limitations (which underlines the need to use technology as noted in the Elderadopt model) and female gender are associated with technology adoption. In an Italian study, Cavallo et al. (2018) revealed that older men and those with higher education relayed higher acceptance of robotic technology and user satisfaction compared to older women and lower education. However, there are studies reporting no gender difference when it comes to robotic acceptance (Fracasso et al., 2022; Naneva et al., 2020). Similar to findings from models presented above (Golant, 2017; Heerink et al., 2010), a focus group study carried out in France with 25 older persons with dementia, their family caregivers, and healthy older persons concluded that persons with dementia and their caregivers perceived higher usefulness of the robot and thus relayed higher intention to use it than healthy older persons (Pino et al., 2015).

With respect to external factors, a pilot project testing a service robot in an assisted living center in the Netherlands reported that the older users responded positively and found the robot to be useful, fun, and safe to use (Portugal et al., 2019). Similarly, Tian, Felber and colleagues’ (2024) systematic review found many positive factors such as providing assistance with basic activities of daily living, reminders for tasks, the potential to affect social isolation, and the formation of new interactions, which may explain robotic technologies’ use or disuse in the care of older adults. Furthermore, negative attitudes toward robots, technology knowledge, skills to use the technology, and infrastructure available negatively influence its uptake, as shown from a study in Sweden (Johansson-Pajala & Gustafsson, 2022) and Switzerland (Felber et al., 2024).

Concerning negative attitudes toward robotic technology, recent empirical and theoretical literature has also raised several ethical concerns (Dequanter et al., 2022; Felber et al., 2023; Wangmo et al., 2019). One that is highly relevant to technology acceptance is the possible replacement of human care (David et al., 2022). That is, acceptance may be a form of not having alternative options, namely humans, to provide necessary care (Vandemeulebroucke et al., 2020). In a similar vein, Fracasso and colleagues (2022) recruited participants from Italy and Germany, and noted that robotic tasks that participants preferred were assistive support, such as holding or grabbing a person, lifting things, cleaning, and reacting to emergent situations, rather than imagining robots as a companion. Other studies with older persons highlighted the importance of human care and tasks deemed acceptable for robots to perform are assistive in nature, such as fetching objects and mowing the lawn (McColl & Nejat, 2013; Park et al., 2019; Vandemeulebroucke et al., 2018, 2021). Different from the above studies underlining the critical value of human care, in an experimental study with MTurkers, Hoppe and colleagues (2023) found that care robots are more often chosen over human caregivers. Only participants who were in poorer health had negative impressions of robots, and were indecisive were more likely to choose human care over care robots.

### Research Questions and Hypotheses

Informed by the literature, this paper assesses the overall acceptance of robots in the care of older persons among a representative sample of the Swiss adult population. We do this by first mapping the acceptance of the four robotic functions examined across different sociodemographic variables. These functions are (a) *companionship*, which was used to highlight robot as partners engaging in listening and talking activities; (b) *doing household chores* included support like cooking, cleaning, and carrying things; (c) *connecting with family members* underscored the ability of a robot to pick up calls and make calls on behalf of the user; and *giving information* included functions of providing information about weather or news to the user. Thereafter, we pose the two research questions (RQ).

RQ 1. Do the functions of robots, as examined in the study, vary with respect to their levels of acceptance?

- H1a: When comparing the two functional groups—“robots for assistive support” and “robots for companionship”—we expect the former to exhibit higher acceptance than the latter. The outcome variable, “robots for assistive support” is the mean of three robotic functions: doing household chores, connecting with family members, and giving information.
- H1b: When comparing “robots for assistive support” and “robots for companionship”—by linguistic region, we predict lower average acceptance for these two functions among German-speaking participants compared to those from the Latin-speaking regions (French- and Italian-speaking regions are combined).
- H1c: Within the functional group “robots for assistive support,” consisting of three items household chores, connecting with family members, and giving information, we expect the following order of acceptance: household chores > giving information > connecting to others.

RQ 2. How can the variance of (a) “robots for assistive support” and (b) “robots for companionship” be explained by different sets of predictors?

- H2a: Set 1 “sociodemographic predictors”—In light of the previous studies underlining the effect of age and gender on technology acceptance, we hypothesize that sociodemographic variables will influence robotic acceptance for both functions.
- H2b. Set 2 “reducing caregiving stress”—As studies underline usefulness as a factor for technology acceptance, perception that robots aid in reducing caregiving stress will lead to greater acceptance of both the robotic functions, controlling for the predictors in set 1. Perceived usefulness was used in the survey as the ability of the robots in helping an older adult with everyday tasks and as a companion, which would contribute toward reducing caregiving stress.
- H2c. Set 3 “positive attitudes toward robots”—Perceptions of finding robots as fun and trustworthy will positively affect acceptance of both robotic functions, controlling for the predictors in sets 1 and 2.
- H2d. Set 4 “negative attitudes toward robots”—For the negative perceptions of having a fear of the robot and fear that robots will replace human care, we expect these to pertain to using “robot for companionship” and not to “robot for assistive support.” That is, these negative attitudes will not affect the function of robots as an assistive tool, controlling for the predictors in sets 1 to 3.

### Rationale for the Study and Research Questions

Shortcomings of the above-mentioned studies, both qualitative and quantitative, often consist of small sample sizes. Furthermore, the systematic review of literature on robotic acceptance does not include representation from Switzerland (David et al., 2022; Naneva et al., 2020; Vandemeulebroucke et al., 2021). Almost two decades ago, Arras and Cerqui (2005) surveyed 2000 persons who visited an exhibition on robotics in Switzerland. They reported an overall good image of robotics, with two-thirds of the participants agreeing that robots could contribute toward their personal welfare, such as supporting with small tasks. However, as the participants were visitors to a robot exhibition, it is possible that they had a greater interest in and a more positive attitude toward robots than the average Swiss population.

Switzerland is an aging society that needs to consider the question of potentially using robotic technologies to care for its growing number of older adults. Furthermore, Switzerland is distinct from other European countries as it is a country with several language regions that are each influenced by different cultures, German vs. Latin (French and Italian). This difference is reflected in the federal votes and termed as the “Röstigraben” (Rey, 2001; Zweimüller, 2018). Differences in opinions among the linguistic regions are found in medical decisions as well (Hendriks et al., 2017; Vilpert et al., 2020). It is likely that the Swiss population may have complex attitudes toward robotic acceptance, which we intend to examine. Therefore, with this study, we present and assess acceptance of robotic technology among a representative sample of adults living in three language regions of Switzerland, in order to test our study hypotheses as well as to present findings from a context from which scarce data is available.

## Method

### Survey Design

We used computer-aided-telephone-interviews (CATI) to carry out the survey of the general population. GFS-Zurich, an established market and social research institute, managed the implementation of the CATI procedure for the study. Together with GFS-Zurich, the first author (principal investigator [PI] of the overall project) agreed on a feasible research implementation plan that constituted a survey duration of approximately 12 min to ensure that participants remain engaged with the topic and motivated to complete the survey.

### Study Sample

In 2021, Switzerland had a population of 8.7 million persons (Federal Statistical Office, 2022b), of which 19% were minors. Taking the adult population as the study population, our sample estimation was 1,056 (margin of error 3%; for a margin of error 5%; sample would have been 387). Published Swiss studies using the general population also tend to have a sample of 1,000 to ensure generalizability (Seifert & Charness, 2022; Sternberg, 2022). For this study, our aim was to capture data from 1,200 adult persons living in Switzerland, a multilingual country where four official languages are spoken (Federal Statistical Office, 2024b).

### Survey Construction

We developed a survey that responds to the goals of our project, as no pre-existing surveys fit. The first version of the survey was based on the preliminary findings of our qualitative interview data obtained from older persons, family caregivers, and professionals in both the German- and French-speaking parts of the country (Felber et al., 2024; Martani et al., 2024; Tian, Duong, et al., 2024; Wangmo et al., 2024). The second source of inspiration for our survey content was the findings of our two systematic reviews on this topic (Felber et al., 2023; [Bibr CIT0039][Bibr CIT0014]). These sets of questions were compared against existing literature on technology acceptance, such as the Elderadopt (Golant, 2017), technology acceptance models (Heerink et al., 2010; Venkatesh et al., 2003) and other literature (Berridge et al., 2022), and fitting questions were adapted and retained for our survey.

### Survey Content

The survey began with demographic information, followed by two questions, which were used as proxies for technology awareness (use of mobile phones and the internet). Relevant to this paper, we asked questions about accepting four robotic functions (household chores, listening and talking, connecting with family members, giving information), which were chosen based on the literature presented above. We also questioned whether the participants would feel that such robotic technology may reduce caregiving stress by supporting household tasks and being a companion based on our literature review (Felber et al., 2023; Tian, Felber, et al., 2024) and findings from the technology models discussed previously. Participants’ attitudes toward acceptance of technology using statements were inspired from a previous study (Heerink et al., 2010) that captured constructs such as perceived enjoyment, anxiety, trust, and facilitating conditions. In response to the recent literature highlighting replacement of human care (Felber et al., 2023; Vandemeulebroucke et al., 2020), we asked two questions about fear of receiving robotic care in particular and fear of replacement of human care in general, a key finding in the ethical analysis of the literature (Felber et al., 2023). We framed these two questions in the same way as the questions adapted from the Almere model (Heerink et al., 2010) used in this survey. Finally, to identify unmet needs based on the work of Golant (2017), we inquired whether they see a need to use the technology for older persons, whether there is enough time to address the needs of an older care receiver, their general physical health status, and if they have or are currently providing care to a family member.

We first vetted the survey internally among the members of the project with backgrounds in gerontology, nursing, political philosophy, statistics, psychology, economics, and bioethics. The project team members included research assistants involved in the collection of qualitative interview data and who participated in the systematic review. Thereafter, we carried out a face validity check by requesting external experts in technology, gerontology, caregiving, and social work to comment on the content of the survey. These experts from both within Switzerland and internationally were purposely selected based on their knowledge of the topic. Either the PI or another member of the research team was familiar with their prior research and thus requested them for support. We received several critical recommendations, which we incorporated. Upon finalization of the survey content in English, independent bilingual research assistants translated the English version into German, French, and Italian. The translated versions of the surveys were back-translated into English, and variations in translations, when noticed, were harmonized. Thereafter, GFS-Zurich piloted the final survey in German with a sample of 30 persons in the general population using the CATI method to ensure that the survey was understandable and time-wise feasible to implement at a larger scale.

### Survey Dissemination

Interviewers from GFS-Zurich disseminated the survey using the CATI method. They used random digit dialing to sample study participants representing the three different linguistic regions of Switzerland: German, French, and Italian. They oversampled the participants from French, and Italian-speaking regions and carried out quota sampling of the population distributed by sex (men and women) and age group (18–39, 40–64, and 65+) to carry out subgroup analysis. The CATI interviews took place between June 8, 2023, and July 10, 2023. The average completion time was 13 min (SD 2.9). GFS-Zurich provided the PI with the data that they had gathered, along with a calculation of sampling weights in accordance with STATPOP (Federal Statistical Office, 2022a), an adult population metric that the Swiss Federal Office of Statistics has provided. Thus, the weights included linguistic regions (71.2% German-speaking, 24.5% French-speaking, and 4.3% Italian-speaking regions). Within the linguistic regions, the proportion of men (49.6%, 48.7%, and 48.3%, respectively) and women (50.4%, 51.3%, and 51.7%, respectively) were also taken into consideration as well as the age group (e.g., for the German-speaking region: 18–39 years: 32.7%; 40–64 years: 41.7%, 65+ years: 25.6%).

### Statistical Analysis

Sampling weights were included in all analyses except for Table 1 where descriptive findings for both weighted and unweighted cases are shown for comparison.

**Table 1.** Sample Characteristics (*N* = 1,211)

Variable	Weighted sample		Unweighted sample	
	*N*	Mean (*SD*) or %	*N*	Mean (*SD*) or %
*Demographic characteristics*				
Age (range: 18–93)	1,211	51.2 (18.18)	1,211	50.8 (18.22)
Age group	1,211		1,211	
18–39 years	413	34.1%	419	34.6%
40–64 years	515	42.5%	520	42.9%
65 years and older	283	23.4%	272	22.5%
Sex	1,211		1,211	
Male	597	49.3%	592	48.9%
Female	614	50.7%	619	51.1%
Marital status	1,195		1,197	
Married	633	52.9%	635	53.0%
Single	347	29.0%	345	28.8%
Divorced	101	8.5%	100	8.4%
Widowed	67	5.6%	66	5.5%
Partnership/cohabiting	48	4.0%	51	4.3%
Linguistic region	1,211		1,211	
German	862	71.2%	606	50.0%
French	297	24.5%	410	33.9%
Italian	52	4.3%	195	16.1%
Educational attainment	1,197		1,200	
Mandatory schooling	109	9.1%	98	8.2%
Apprenticeship	492	41.1%	503	41.9%
High school	111	9.3%	136	11.3%
University	485	40.5%	463	38.6%
*Caregiving Situation*				
Enough time to care for an older family member?	1,182		1,175	
Definitely no	131	11.1%	139	11.8%
Probably no	327	27.7%	319	27.1%
Maybe	214	18.1%	198	16.9%
Probably yes	255	21.6%	269	22.9%
Definitely yes	254	21.5%	250	21.3%
Need to use new technologies	1,181		1,182	
Definitely no	310	26.2%	329	27.8%
Probably no	242	20.5%	251	21.2%
Maybe	285	24.1%	268	22.7%
Probably	229	19.3%	231	19.5%
Definitely yes	116	9.8%	103	8.7%
Self-rated health	1,203		1,203	
Poor/fair	54	4.5%	73	6.1%
Good	490	40.8%	508	42.2%
Very good	410	34.1%	393	32.7%
Excellent	248	20.7%	229	19.0%
Caregiver for an older family member?	1,206		1,207	
No, not a carer	788	65.4%	769	63.7%
Yes, a caregiver	418	34.6%	438	36.3%
*Tech awareness*				
Using mobile phones	1,210		1,208	
Not/somewhat not familiar^a^	95	7.9%	91	7.5%
Neither nor	78	6.4%	87	7.2%
Somewhat familiar	480	39.7%	482	39.9%
Very familiar	556	46.0%	548	45.4%
Using internet	1,211		1,211	
Beginner/do not use^a^	111	9.2%	110	9.1%
Average	374	30.9%	383	31.6%
Competent	399	33.0%	392	32.4%
Very competent	327	27.0%	326	26.9%

^a^Two cells combined as responses were less than 5% in each cell.

To respond to the first research question, we conducted a descriptive analysis to map the key characteristics of the study sample alongside their acceptance levels of four robotic functions: (a) robots for household tasks, (b) robots as a companion, that is, listening and talking; (c) robots for connecting with others; and (d) robots for giving information. Acceptance was measured using a Likert scale with a score of 1–5, with 5 indicating the highest acceptance (very acceptable) and 1 indicating the lowest acceptance (not acceptable at all).

To test the hypotheses for RQ 1 (H1a–c), we employed a multilevel modeling approach. We checked for multicollinearity in the data using the variance inflation factor (VIF) and found no problems in this respect (all VIF values were between 1 and 1.6, which is far below the threshold of 10 or higher). Multilevel models account for the dependency in the data as a result of measuring four different robotic functions on the same individual. The multilevel model contained a random intercept and specific contrasts for comparing acceptance levels across different robotic functions. H1a thereby tested the contrast that the acceptance of (a) “robots for assistive support” (i.e., the combined three robotic functions, doing household chores, giving information, connecting with others) was higher than that for (b) “robots for companionship” (i.e., the recoded function robot for talking and listening purposes). H1b was based on the same model as H1a but included, in addition, the predictor linguistic region, whereby the Italian and French regions were lumped together to become “Latin languages.” This hypothesis tested whether the acceptance of these two robotic functions was lower in the German language region compared to the Latin language region. Finally, H1c tested the linear contrast that within “robots for assistive support” the acceptance increased in the following order: household chores > giving information > connecting to others.

To understand the acceptance of robotic technology as an assistive support and for companionship, in the care of older persons, we conducted an explorative analysis in hypothesis 2 (H2a–d), thereby using sequential multiple regression analysis. This model consisted of four different sets of predictors that were entered sequentially into our statistical model. The order in which these sets were entered was based on our best judgment of the available literature: (a) sociodemographic variables, which included age, gender, education, marital status; (b) usefulness of robotic functions to reduce caregiving stress by supporting with household chores or being a companion; (c) facilitating conditions that allow uptake of robotic technology such as finding it fun and trusting the robot; and (d) hindering factors like fear of robots and fear of replacement of human care. The explained variance for a particular set of predictors was thereby always controlled for predictors of sets being entered before that set but not controlled for predictors of sets being entered after that set. This model was carried out separately for the two outcomes, “robots for assistive support” and “robots for companionship.”

All analyses were carried out using the statistical software packages SPSS (version 29.0) and R (version 4.4.1), including the R-packages survey (version 4.4-2) and lme4 (version 1.1-35.5).

## Results

### Sample Characteristics

A total of 1,211 adults living in Switzerland participated in this study. As per our sampling strategy, we oversampled participants from the French- and Italian-speaking regions, resulting in exactly half of the sample (*n* = 606) from the German-speaking region, and the remaining were recruited from the French-speaking (*n* = 410) and Italian-speaking (*n* = 195) regions (see Table 1). In order to generalize the findings to the Swiss adult population, the results presented are weighted by linguistic region, gender, and age to mimic the general adult population (Federal Statistical Office, 2022a). Thus, after weighing, the sample constitutes 71.2% (*n* = 862) from German-speaking Switzerland, 24.5% (*n* = 297) from the French-speaking region, and 4.3% (*n* = 52) from the Italian-speaking region (see Table 1). In addition, the sample is weighted to be 50.7% female. Both weighted and unweighted sample characteristics (e.g., age, gender, education, marital status) are presented in Table 1.

In addition to demographics, we also inquired about the participants’ care situation. About 40% of the sample reported not having enough time to care for an older family member (see Table 1). When questioned if they see the need to use new technologies in their current situation, 29.1% responded affirmatively. A third of the participants (34.6%) revealed that they are a caregiver to an older family member, and almost all of the participants (95.5%) reported being in good, very good, or excellent health.

Concerning their technology awareness, participants responded to two questions related to their familiarity with mobile phones and the internet. On a scale from 1 to 5 (Table 1), 85.7% of the respondents were positive (very comfortable = 5, somewhat comfortable = 4) about using mobile phones (mean 4.2, median 4). Among the younger ages (18–64 years), only 2.4% reported being not familiar with using mobiles, whereas the proportion was 13.2% among the oldest group (65+). In the case of internet usage, about 60.0% reported being competent or very competent (mean 3.7, median 4). As expected, among the younger ages (18–64 years), only 7.0% reported to be beginners or not using the internet, whereas the proportion among older persons (65 years and older) was 20.1%.

Table 2 provides descriptive information on the acceptance of the four robotic functions (household tasks, listening and talking, connecting with others, and giving information) by participants’ sociodemographic characteristics, their health status, and their care situation.

**Table 2.** Weighted Acceptance^a^ of Robotic Functions by Participant Characteristics (*N* = 1211)

Variable	Household chores			Listening and talking			Connecting to others			Giving information		
	Mean	*SD*	Median	Mean	SD	Median	Mean	SD	Median	Mean	SD	Median
Age group (*N* = 1,211)												
18–39 years (*n* = 413)	3.63	1.18	4	2.72	1.31	3	3.83	1.10	4	3.59	1.18	4
40–64 years (*n* = 515)	3.21	1.55	3	2.70	1.51	3	3.61	1.51	4	3.28	1.55	4
65+ years (*n* = 283)	2.63	1.60	2	2.31	1.57	1	2.89	1.68	3	2.83	1.70	3
Sex (*N* = 1,211)												
Male (*n* = 597)	3.49	1.41	4	2.80	1.44	3	3.70	1.35	4	3.49	1.43	4
Female (*n* = 614)	2.95	1.52	3	2.43	1.47	2	3.34	1.56	4	3.07	1.54	3
Marital status (*N* = 1,195)												
Married (*n* = 633)	3.13	1.51	3	2.53	1.48	2	3.44	1.52	4	3.22	1.53	3
Single (*n* = 347)	3.53	1.36	4	2.83	1.41	3	3.75	1.25	4	3.53	1.38	4
Divorced (*n* = 101)	3.09	1.56	3	2.78	1.62	3	3.61	1.60	4	3.28	1.61	4
Widowed (*n* = 67)	2.49	1.64	2	2.42	1.52	2	3.00	1.81	3	2.93	1.74	3
Partnership/cohabiting (*n* = 48)	3.56	1.10	4	2.31	1.15	2	3.70	1.15	4	3.17	1.02	3
Linguistic region (*n* = 1,211)												
German (*n* = 862)	3.22	1.48	3	2.61	1.47	2	3.59	1.47	4	3.30	1.49	4
French (*n* = 297)	3.26	1.54	4	2.64	1.49	3	3.31	1.47	3	3.24	1.52	3
Italian (*n* = 52)	2.98	1.49	3	2.54	1.46	2	3.62	1.46	4	3.11	1.48	3
Educational attainment (*N* = 1,197)												
Mandatory schooling (*n* = 109)	2.42	1.55	2	2.47	1.62	2	2.89	1.63	3	2.84	1.63	3
Apprenticeship (*n* = 492)	3.08	1.41	3	2.46	1.37	2	3.42	1.43	4	3.11	1.40	3
High school (*n* = 111)	3.19	1.55	3	2.56	1.46	3	3.39	1.50	3	3.07	1.56	3
University (*n* = 485)	3.57	1.44	4	2.83	1.50	3	3.81	1.40	4	3.62	1.48	4
Enough time to care for an older family member? (*N* = 1,182)												
Definitely no (*n* = 131)	3.26	1.47	4	2.67	1.44	3	3.37	1.43	4	3.19	1.50	3
Probably no (*n* = 327)	3.55	1.31	4	2.70	1.34	3	3.85	1.29	4	3.57	1.26	4
Maybe enough time (*n* = 214)	3.34	1.43	4	2.62	1.52	2	3.65	1.33	4	3.41	1.43	4
Probably yes (*n* = 255)	3.19	1.51	3	2.74	1.53	3	3.63	1.49	4	3.34	1.59	4
Definitely yes (*n* = 254)	2.67	1.61	2	2.30	1.53	2	3.01	1.68	3	2.80	1.66	3
Seeing a need to use new technologies? (*N* = 1,181)												
Definitely no (*n* = 310)	2.82	1.57	3	2.11	1.28	2	2.89	1.51	3	2.72	1.49	3
Probably no (*n* = 242)	2.92	1.45	3	2.28	1.29	2	3.28	1.48	4	3.07	1.43	3
Maybe (*n* = 285)	3.27	1.31	3	2.67	1.45	3	3.68	1.35	4	3.31	1.40	4
Probably yes (*n* = 229)	3.53	1.49	4	3.21	1.50	3	4.05	1.25	5	3.89	1.37	4
Definitely yes (*n* = 116)	4.14	1.28	5	3.41	1.59	4	4.27	1.28	5	4.08	1.37	5
Self-rated health (*N* = 1,203)												
Poor self-rated health (*n* = 54)	2.94	1.57	3	2.73	1.63	3	3.34	1.54	3	3.07	1.62	3
Good (*n* = 490)	3.11	1.41	3	2.50	1.44	2	3.47	1.50	4	3.13	1.47	3
Very good (*n* = 410)	3.35	1.47	4	2.72	1.44	3	3.57	1.40	4	3.41	1.41	4
Excellent (*n* = 248)	3.31	1.62	4	2.67	1.54	3	3.60	1.51	4	3.44	1.54	4
Are you a caregiver for an older family member? (*N* = 1,206)												
No, not a carer (*n* = 788)	3.38	1.40	4	2.70	1.42	3	3.63	1.36	4	3.37	1.40	4
Yes, a caregiver (*n* = 418)	2.94	1.61	3	2.46	1.56	2	3.34	1.63	4	3.14	1.65	3
How comfortable are you using mobile phones? (*N* = 1,210)												
Not/somewhat not familiar (*n* = 95)^b^	2.64	1.72	2	2.27	1.57	1	3.15	1.71	3	2.64	1.73	2
Neither nor (*n* = 78)	2.92	1.59	3	2.59	1.58	2	3.17	1.67	3	2.94	1.56	3
Somewhat familiar (*n* = 480)	3.03	1.40	3	2.41	1.36	2	3.48	1.40	4	3.17	1.36	3
Very familiar (*n* = 556)	3.52	1.46	4	2.85	1.50	3	3.67	1.44	4	3.54	1.51	4
How would you describe yourself in regards to the use of internet? (*N* = 1,211)												
Beginner/do not use^b^ (*n* = 111)	2.72	1.48	3	2.40	1.44	2	2.99	1.60	3	2.86	1.56	3
Average (*n* = 374)	2.90	1.47	3	2.33	1.43	2	3.41	1.52	4	3.06	1.45	3
Competent (*n* = 399)	3.15	1.45	3	2.55	1.45	2	3.44	1.46	4	3.15	1.52	3
Very competent (*n* = 327)	3.83	1.38	4	3.08	1.45	3	3.92	1.29	4	3.83	1.37	4

^a^Acceptance level (1–5): 1 = lowest acceptance, 5 = highest acceptance.

^b^Two cells combined as responses were less than 5% in each cell.

### Hypothesis RQ1—Comparing Acceptance Levels Among Different Robotic Functions

Descriptive values of the four robotic functions—household chores, listening and talking, connecting to others, and giving information—are shown in Table 3. Acceptance was higher for “robots for assistive support” (i.e., the combined three robotic functions: household chores, connecting to others, and giving information) than for “robots for companionship” (see H1a), with the mean differences between the two groups being 0.708 (*SE* = 0.031, *z* = 22.9, *p* < .001). Mean acceptance for the combined functions “robots for assistive support” and “robots for companionship” was 3.19 (*SE* = 0.049) for the German language region, and 3.10 (*SE* = 0.054) for the Latin language region and did not differ between the two language regions (*b* = 0.092, *SE* = 0.073, *z* = 1.26, *p* = .206, see H1b). Finally, within “robots for assistive support,” acceptance levels linearly increased across the three functions—household chores, giving information, and connecting to others (*b* = 0.152, *SE* = 0.019, *p* < .001, see H1c), a progression that was different from what we hypothesized.

**Table 3.** Weighted Acceptance of Technology Functions

Technology functions	Mean of acceptance^a^ (*SD*)	Percentage of acceptance (%)^b^	P25, P50, P75
Household chores (*N* = 1,204)	3.21 (1.49)	49.50	2.0, 3.0, 5.0
Listening and talking (*N* = 1,190)	2.61 (1.47)	28.80	1.0, 2.0, 4.0
Connecting to others (*N* = 1,204)	3.52 (1.47)	57.40	3.0, 4.0, 5.0
Giving information (*N* = 1,207)	3.28 (1.50)	49.00	2.0, 3.0, 5.0

^a^Acceptance level (1–5): 1 = lowest acceptance, 5 = highest acceptance.

^b^Percentage of acceptance calculated using those who answered “somewhat acceptable” and “very acceptable,” that is, choose 4 and 5. Other response choices were 3 “neither acceptable nor unacceptable,” 2 “somewhat unacceptable,” and 1 “not acceptable at all.”

### Hypothesis RQ 2—Explanation of Acceptance of Robotic Functions

Sequential multiple regression analysis revealed that though all four sets of predictors accounted for the variance of “robots for assistive support” and “robots for companionship” to some degree, the second set, “usefulness of robotic functions” to reduce caregiving stress by either (a) supporting with household chores or (b) through being a partner, was by far the most important one. Results are shown in Table 4. The first and third sets, “sociodemographics” and “finding robots fun and trusting robot,” respectively, also explained a certain amount of variance, albeit much less than the second set. The fourth set explained the least amount of variance, especially for the outcome “robots for assistive support.”

**Table 4.** Impact of Four Sets of Predictors on the Two Outcome Functions “Robots for Assistive Support” and “Robots for Companionship,” Using Sequential Multiple Regression

Variable	Model fit (pseudo *R*^2^)	Model improvement (change in pseudo *R*^2^)	Change in deviance	*p* Value^a^
*Outcome: “robots for assistive support”*				
Null model	0	—	—	—
(1) Sociodemographics (age, gender, education, marital)	0.122	0.122	122.7	<.001
(2) Usefulness of robotic functions to reduce caregiving stress	0.581	0.459	740.8	<.001
(3) finding robots fun and trusting robot	0.598	0.017	28.9	<.001
(4) hindering factors like fear of robots and fear of replacement of human care	0.601	0.004	5.8	.055
*Outcome: “robots for companionship”*				
Null model	0	—	—	—
(1) Sociodemographics (age, gender, education, marital)	0.040	0.040	36.9	<.001
(2) Usefulness of robotic functions to reduce caregiving stress	0.403	0.363	499.8	<.001
(3) finding robots fun and trusting robot	0.429	0.027	33.9	<.001
(4) hindering factors like fear of robots and fear of replacement of human care	0.438	0.009	10.2	.007

^a^ *p* Values relate to the improvement in the model fit (change in deviance) when including the respective set of predictors.

## Discussion and Implications

Our analysis of the data using weighted samples allows us to generalize the findings for the Swiss adult population as our sample represented this group by linguistic region, age group, and gender (Federal Statistical Office, 2022a). Additionally, two-fifths (40.5%) of the sample reported having attended university education (Bachelor education or higher), and 41.1% possessed apprenticeship education, which somewhat mirrors the educational status of the Swiss population (Federal Statistical Office, 2024a). As expected, among those categorized as younger ages in our sample (18–64 years), only about 7% reported to be beginners or not using the internet, whereas the proportion among older persons (65 years and older) was 20.1%. A recent study on digital literacy noted that digital literacy decreases with increasing age, with the youngest age-group (18–39 years) assessing themselves as more digitally savvy compared to those who were 65 years and older (Sternberg, 2022). Other studies among the older Swiss population highlighted that only between 9% and 20% are noninternet users (König & Seifert, 2023; Seifert & Charness, 2022).

From our study results, lower acceptance was found for using robots as a companion than for using robots for assistive support. The result is in accordance with hypothesis 1a, that viewing robots as an entity to be treated as a friend would be less accepted compared to robotic functions that are practical in nature (Arras & Cerqui, 2005; Fracasso et al., 2022; Vandemeulebroucke et al., 2020). Among the three assistive functions examined, we predicted the highest acceptance for “household chores” based on previous studies (Fracasso et al., 2022; McColl & Nejat, 2013; Park et al., 2019). However, “connecting with others” and “giving information” had a higher acceptance score than supporting with “household chores.” This may also be related to the perspectives relayed in qualitative studies, where, despite the need for technology support, older adults still want to do as much as possible by themselves and not risk becoming dependent (Coghlan et al., 2021). Finally, for hypothesis 1b, although we expected to see differences amongst the linguistic regions, specifically with lower acceptance among those living in the German-speaking part compared to the Latin-speaking parts (Rey, 2001; Zweimüller, 2018), our analysis showed no such statistical difference. This could be an indication that instead of linguistic regions, a more important factor could be the differences between rural and urban settings, which we did not test for.

Similar to previous studies on technology acceptance (Cavallo et al., 2018; Heerink et al., 2010; Huang & Oteng, 2023; Venkatesh et al., 2003), in the second research question, we hypothesized that age, gender, and education have an effect on technology acceptance. From our sequential multiple regression analysis, we found that sociodemographic characters—age, gender, marital status and education—had a significant effect on both the models explaining 12% of the outcome “robots for assistive support” and 4% of the outcome “robots for companionship,” thereby indicating that technology acceptance is explained by these predictors.

Golant (2017) predicted that the greater needs for support of older persons due to, for example, poor health, having less time to care for an older person, and being a caregiver will lead to greater technology acceptance. In addition, if a technology is deemed to be useful (Golant, 2017; Heerink et al., 2010; Pino et al., 2015), the acceptance of the technology will increase. Correspondingly, we find that for both functions “robots for assistive support” and “robots for companionship,” the perception that using them will reduce caregiving stress had the highest impact on the model (46% and 36% variance explained, respectively). Although this positive direction of the finding was expected, its strength found was not expected. This was a surprising finding, as our key hypotheses were based on the external factors (e.g., fun and fear) involved in technology acceptance. One can nevertheless argue that the actual usefulness of the technology in the care space might be a core motivation to technology acceptance, which is in line with what we know from previous literature (Golant, 2017; Thielke et al., 2012).

We expected a high impact from the external factors like fun, trust, and, most importantly, fear that we included in our model (Heerink et al., 2010; Pino et al., 2015; Portugal et al., 2019; Venkatesh et al., 2003). Positive perceptions (i.e., fun and trust) were significant in explaining the acceptance of the two robotic functions, but the additional variance they explain was very low (less than 3% for both outcome variables). Most importantly, in the case of the fear of robotic technology itself and its potential to replace human care (Felber et al., 2023; Wangmo et al., 2019), we find the least variance explained for both models. In the case of using “robots for assistive support” as expected in our hypothesis, fear of technology and fear that technology will replace human care was not significant. We underline that in the function of assistive support, we did not include basic support such as showering and eating assistance, where substitution by a robot may face greater resistance.

Only for using “robots for companionship,” the findings for both predictors of fear were significant at *p* < .05 level. However, the additional variance explained remained very low. We underline here that in the sequential multiple regression model, we included this set as the fourth level, which controls for all the previous sets and since all predictors are likely to be related to one another, there may be very little left to explain by the two fear variables. At the same time, it could be that we placed more expectations on the external factors in our hypothesis based on studies that have repeatedly warned against the possible reduction of human care, a narrative that has become quite pervasive (Parks, 2010; Sharkey & Sharkey, 2012; Sparrow & Sparrow, 2006). Hoppe and colleagues (2023) in their experimental study have shown that the care robot was chosen more often than the human caregiver. Our findings provide some support for this notion that the importance placed on human care may not be as significant as we wish to believe.

### Limitations

Previous studies have reported that the acceptability level improves with actual technology interaction and knowledge compared to when there was no interaction or no prior knowledge (David et al., 2022; Naneva et al., 2020). First, in our survey of the general adult population, we did not include an interaction with the actual technology. We assumed that the participants would have some familiarity with technologies in general, confirmed by the technology awareness indicators that we have from our sample. Second, we adapted several existing questions from acceptance models (Davis, 1987; Heerink et al., 2010; Venkatesh et al., 2003) and developed questions based on preliminary analysis of our published works (Felber et al., 2023, 2024; Martani et al., 2024; Tian, Duong, et al., 2024; Wangmo et al., 2024). Hence, our questions were not tested for psychometric values to underline the construct validity and reliability. Third, in this paper, we limited our predictors to what we deemed as most important based on the state of the art and what is feasible for one publication. We acknowledge that our study focused on the acceptance of only four types of robotic functions (household chores, talking and listening, connecting with others, giving information), which is a simplification as the care of older persons includes other tasks and skills that we did not cover. If more advanced care functions (e.g., clinical assessment using AI) had been included in the study, the results would probably have been different. Fourth, our sequential multiple regression included a set of predictor variables whose order was determined based on our best judgment of the available literature presented in the introduction. One may question the order of the sequence. Finally, the telephone survey method comes with its own limitations. For example, in terms of equity and diversity, our recruitment method did not seek to recruit participants with communication, hearing, and other barriers (such as dementia), which calls for future studies with these groups. Additionally, the limited time we had to complete the telephone survey and the risk of participant fatigue meant that we could not provide extensive definitions for the terminologies used in the questions. Our attempt was to keep the questions as clear as possible to limit variations in understanding. As an example, the burden of care question was framed as follows: On a scale of 1–5, with 1 being “not useful at all” and 5 being “very useful,” please indicate how useful the following technologies would be, in your opinion in reducing caregiving stress: “robots that help an older family member with everyday tasks” and “robots as a conversation partner for an older family member.”

### Implications and Future Research

Among our survey respondents, we find greater acceptance of robots when their use was deemed for assistance purposes rather than as companions. This finding calls for differentiating how much and which care can be outsourced to robots and those that should be performed by caregivers only. Also, it relays the need to critically evaluate whether the values placed on human care are or should be a negotiable factor when supporting older persons. We learn that robotic technologies were accepted if it was perceived to reduce caregiving stress for the caregivers. Therefore, the means to efficiently place robotic technology in the care space where such perceived and felt care burdens can be reduced is critical. The establishment of the household care routine with robots stands to fill the care situation of older adults and support caregivers as well. However, care support needed will evolve with the development of technology, the changing needs of older care recipients, as well as the varying potential of the caregivers. In the same vein, there will be those who refuse the use of such technologies despite their potential as useful tools. Refusals can be for many reasons, ranging from personal to social to individual ability to afford the technology. At the same time, there is a danger that, in the future (when robotic use may be normalized and cost issues resolved), refusals may only be reserved for those who are more privileged. Thus, it becomes critical to continuously study the use of robotic technology in the support of older persons as well as monitor how such uses may be shaping the society.

## Conclusions

The adult general population from Switzerland revealed lower acceptance for robots when used as a companion in social activities compared to using robots as an assistive tool to support with practical chores. From our results, it appears that the perception of robot’s usefulness in ameliorating caregiving stress and individual characteristics have much greater impact on technology acceptance than external factors like perceiving it to be fun, trusting it, and even fearing the robot itself or the consequences its use may bring, that is, replace human care. Future research is needed to further test the impacts of external factors relevant for robotic acceptance in particular, as well as technology adoption or rejection in general with diverse groups of older adults and their caregivers.

## Data Availability

The survey dataset, from which the paper stems, will be made available on SWISSUBase, an Open Access Platform, in December 2025.
